# From obesity to healthy longevity: a consensus-based clinical framework for diagnosis, staging, and treatment

**DOI:** 10.3389/fragi.2026.1834323

**Published:** 2026-08-14

**Authors:** Silvija Canecki-Varžić, Ines Bilić-Ćurčić, Maja Bakula, Tomislav Bulum, Maja Čavlović, Diana Delić-Brkljačić, Tamara Turk Wensveen, Daniela Fabris-Vitković, Dubravka Jurišić-Eržen, Sanja Klobučar, Kristina Kljajić, Mladen Krnić, Blaženka Miškić, Nataša Moser, Božidar Novoselović, Ivan Pećin, Tina Tičinović-Kurir, Jelena Vučak-Lončar, Marina Gradišer, Maja Cigrovski Berković, Gorana Mirošević, Kristina Selthofer-Relatić, Miro Bakula

**Affiliations:** 1 Department of Endocrinology, Clinic of Internal Medicine, Osijek University Hospital Centre, Osijek, Croatia; 2 Faculty of Medicine Osijek, Josip Juraj Strossmayer University of Osijek, Osijek, Croatia; 3 Faculty of Dental Medicine and Health Osijek, Josip Juraj Strossmayer University of Osijek, Osijek, Croatia; 4 Department of Diabetes and Endocrinology, University Clinic for Diabetes, Endocrinology and Metabolic Diseases Vuk Vrhovac, Merkur University Hospital, Zagreb, Croatia; 5 Faculty of Medicine, University of Zagreb, Zagreb, Croatia; 6 Clinic for Cardiovascular Diseases, Sestre Milosrdnice University Hospital Centre, Zagreb, Croatia; 7 Faculty of Medicine, University of Rijeka, Rijeka, Croatia; 8 Center for Endocrinology, Diabetes and Cardiometabolism, Clinic for Treatment, Rehabilitation and Prevention of Cardiovascular Diseases, Thalassotherapia Opatija, Opatija, Croatia; 9 Department of Endocrinology and Diabetology, Pula General Hospital, Pula, Croatia; 10 Faculty of Medicine, Juraj Dobrila University of Pula, Pula, Croatia; 11 Department of Endocrinology, Diabetes and Metabolic Diseases, Rijeka University Hospital Centre, Rijeka, Croatia; 12 Department of Endocrinology, Diabetology and Metabolic Disorders, Split University Hospital Centre, Split, Croatia; 13 Faculty of Medicine, University of Split, Split, Croatia; 14 Josip Benčević General Hospital, Slavonski Brod, Croatia; 15 Department of Endocrinology, Diabetes and Metabolic Diseases “Mladen Sekso”, Sestre Milosrdnice University Hospital Centre, Zagreb, Croatia; 16 Department of Metabolic Diseases, Zagreb University Hospital Centre, Zagreb, Croatia; 17 Department of Endocrinology, Diabetes and Metabolic Diseases, Department of Internal Medicine, Zadar General Hospital, Zadar, Croatia; 18 Department of Health Studies, University of Zadar, Zadar, Croatia; 19 Department of Internal Medicine, Čakovec County Hospital, Čakovec, Croatia; 20 Faculty of Kinesiology, Department for Sport and Exercise Medicine, University of Zagreb, Zagreb, Croatia; 21 School of Dental Medicine, University of Zagreb, Zagreb, Croatia; 22 Department for Heart and Vascular Diseases, Clinic of Internal Medicine, Osijek University Hospital Centre, Osijek, Croatia; 23 Department of Endocrinology, Diabetes and Metabolic Diseases, Sveti Duh University Hospital, Zagreb, Croatia

**Keywords:** aging, bariatric surgery, comorbidity, Croatia, disease staging, healthy longevity, obesity, pharmacotherapy

## Abstract

**Background:**

Obesity is a chronic, progressive, relapsing disease characterized by excess or dysfunctional adiposity that impairs health and contributes to multimorbidity, disability, and reduced life expectancy. Contemporary international frameworks recommend moving beyond BMI-only definitions toward disease-based diagnostic models that integrate adiposity, complications, and functional status. Croatia faces a high prevalence of obesity alongside constrained healthcare resources and limited reimbursement for modern pharmacotherapy, highlighting the need for nationally adapted clinical guidance.

**Methods:**

This position statement was developed using a modified Delphi consensus process involving 23 multidisciplinary experts from academic and clinical institutions across Croatia. Consensus statements were derived from contemporary international guidelines, randomized controlled trials, and meta-analyses. Evidence quality and recommendation strength were graded using an adapted GRADE-based framework.

**Results:**

The document establishes a Croatia-specific framework for the diagnosis, staging, and management of obesity. Diagnosis requires confirmation of excess adiposity with evidence of clinical risk or dysfunction and integrates anthropometric measures with metabolic, mechanical, psychological, and functional domains. A staging system aligned with EASO and Lancet frameworks guides treatment intensity according to disease severity. Management is based on four pillars: lifestyle therapy, psychological support, pharmacotherapy, and metabolic/bariatric surgery. Pharmacological treatment is stage-based and comorbidity-driven, prioritizing agents with proven organ-protective effects. Special considerations are provided for older adults, emphasizing preservation of muscle mass, functional capacity, independence, and quality of life. Long-term structured follow-up is recommended for all patients, reflecting the chronic disease model of obesity.

**Conclusion:**

This position statement provides an evidence-based, nationally adapted clinical framework that aligns obesity management with modern chronic disease standards. By integrating disease staging, individualized therapy, and long-term care, it supports precision treatment across the lifespan and emphasizes obesity management as a key strategy for preventing complications, reducing disability, and promoting healthy longevity.

## 1 Scope and purpose of this statement

This position statement provides an evidence-based, clinically oriented framework for the diagnosis, staging, and management of obesity across the adult lifespan, with emphasis on prevention of complications, preservation of functional capacity, and promotion of healthy longevity. It translates contemporary international scientific recommendations into a structured model designed for real-world clinical practice and healthcare systems with varying levels of resources.

The document is intended for healthcare professionals involved in obesity care, including endocrinologists, diabetologists, internal medicine specialists, primary care physicians, bariatric and metabolic surgeons, dietitians, psychologists, physiotherapists, and multidisciplinary teams, as well as policymakers and healthcare administrators responsible for implementing clinical pathways and chronic disease strategies.

Its objectives are threefold: (1) to support individualized, stage-based clinical decision-making across lifestyle, pharmacological, surgical, and long-term management approaches; (2) to outline integrated care pathways spanning primary, secondary, and tertiary levels; and (3) to inform health-system planning and policy development related to obesity as a chronic disease.

These recommendations are intended as a consensus-based reference framework rather than prescriptive rules. Clinical judgment, patient priorities, comorbidity burden, and local healthcare context should guide final decisions. By integrating disease staging, multidisciplinary therapy, perioperative optimization, and lifelong follow-up, this statement supports a chronic disease model of obesity care aimed at improving outcomes, reducing disability, and enhancing long-term health and independence**.**

## 2 Executive summary

Obesity is a chronic, progressive, relapsing disease characterized by excess adiposity that impairs health (Busetto et al., 2024; Guidelines, 2025; Rubino et al., 2025; De Luca et al., 2024; Grunvald et al., 2022). The latest international frameworks (Lancet Commission 2025; EASO 2025 Delphi consensus, NICE 2025 guideline) call for redefining obesity diagnosis beyond BMI alone and for aligning management with the standards applied in other chronic diseases (Busetto et al., 2024; Guidelines, 2025; Rubino et al., 2025; De Luca et al., 2024; Grunvald et al., 2022).

Croatia faces high obesity prevalence and limited healthcare resources. Modern obesity pharmacotherapy (GLP-1/GIP receptor agonists) is available but currently not reimbursed for obesity by Croatian National Health Fund, creating inequities in access (De Luca et al., 2024; Grunvald et al., 2022). Orlistat remains the only broadly reimbursed option (Finer et al., 2000; Torgerson et al., 2004). This statement provides a Croatia-specific framework for diagnosis, staging, and management of obesity, with stratification by disease stage, comorbidities, and treatment priorities (Busetto et al., 2024; Guidelines, 2025; Rubino et al., 2025; De Luca et al., 2024; Grunvald et al., 2022). Particular attention is given to aging populations, in whom obesity contributes to functional decline, multimorbidity, and loss of independence, requiring diagnostic and therapeutic approaches that prioritize healthy longevity and preservation of physical function (Di Vincenzo et al., 2025). Pharmacological recommendations are aligned with the European Association for the Study of Obesity (EASO) framework and the World Health Organization (WHO) guideline on the use of GLP-1–based therapies for obesity, emphasizing stage-based, comorbidity-driven treatment within a chronic disease care model (McGowan et al., 2025; World Health Organization, 2025).

## 3 Methods

### 3.1 Delphi consensus methodology

This position statement was developed using a modified Delphi consensus process, designed to achieve structured expert agreement in areas of evolving evidence and national contextual relevance. A panel of 23 experts was assembled, representing endocrinology, diabetology, internal medicine, obesity medicine, cardiology, kinesiology, and related disciplines. The panel was intentionally composed of clinicians directly involved in diagnosis and prescribing decisions within the Croatian healthcare system, where pharmacological and surgical decisions are physician-led. Panelists were recruited from university hospitals, regional hospitals, and academic institutions across Croatia, ensuring broad geographic and professional representation. Participation was voluntary and anonymous. An initial set of 31 consensus statements was developed by the steering group based on: contemporary international guidelines and consensus documents (Lancet Commission 2025; EASO 2024/2025, NICE 2025), high-quality randomized controlled trials and meta-analyses, and gaps identified in Croatian clinical practice and healthcare organization. Statements were grouped into seven domains: conceptual definition of obesity; diagnostic criteria and screening; treatment principles; pharmacotherapy; bariatric/metabolic surgery; health system and policy. Panelists rated each statement on a nine-point Likert scale (1–3 = Disagree; 4–6 = Uncertain; 7–9 = Agree). Free-text comments were encouraged to capture qualitative feedback and suggestions for refinement. Consensus thresholds were defined *a priori*: strong consensus: ≥80% of ratings in the seven to nine range; moderate consensus: 60%–79% of ratings in the seven to nine range; no consensus: <60% of ratings in the seven to nine range.

In Round 1, the expert panel evaluated 31 statements addressing the definition, diagnosis, staging, and management of obesity. Strong consensus (≥80% agreement) was achieved for the majority of statements. A small subset of statements demonstrated moderate consensus (60%–79%), primarily related to: the diagnostic formulation of obesity in the absence of overt organ dysfunction, recommended frequency of follow-up, criteria for modification of pharmacotherapy after suboptimal response, and indications for metabolic/bariatric surgery at lower BMI thresholds. Qualitative feedback highlighted the need for clearer distinction between preclinical and clinical obesity, greater flexibility in follow-up intervals, explicit reassessment before treatment escalation, and stricter patient selection criteria for surgery at lower BMI. These statements were revised accordingly and re-evaluated in Round 2. Following incorporation of Round one results and expert feedback, a second Delphi round was conducted. In Round 2, all participating experts rated every revised statement within the agreement range (scores 7–9 on the nine-point Likert scale). Consensus was defined *a priori* as ≥80% of panelists rating a statement within the agreement range (scores 7–9). The term ‘100% agreement’ in Round two indicates that all responding panelists rated each statement within this predefined agreement range, although individual scores were not necessarily identical. Response rates were 100% in both rounds, and all panelists participated in both iterations. No statements required further modification, and no additional Delphi rounds were necessary. Consensus was therefore formally achieved after two Delphi rounds.

The Delphi process was conducted using anonymized electronic questionnaires administered centrally by the steering group. Individual responses were not visible to other panelists at any stage, and no direct interaction between panelists occurred during the rating process.

The finalized statements represent the collective expert agreement of the panel and form the evidentiary and conceptual foundation of this position statement on the diagnosis, staging, and management of obesity in Croatia (Table 1).

**TABLE 1:** Final delphi consensus statements with evidence grading and strength of recommendation.

Domain	No.	Final delphi statement (exact questionnaire wording)	Quality of evidence	Strength of recommendation
Conceptual Definition	A1	Obesity is a chronic, relapsing, systemic disease caused by excess or abnormal adiposity that impairs health	High	Strong
	A2	BMI is a screening tool, not a diagnostic endpoint	High	Strong
	A3	Diagnosis of obesity requires evidence of excess or abnormal adiposity, with stratification into preclinical obesity (increased health risk without overt organ dysfunction) and clinical obesity (with established obesity-related complications or dysfunction)	High	Strong
	A4	Adopt the Lancet 2025 framework: Preclinical vs. Clinical Obesity	Moderate	Strong
	A5	Use EASO 2024/2025 staging (0–3) to determine disease severity	Moderate	Strong
Diagnostic Criteria and Screening	B1	Use WHO BMI cut-offs; apply Asian thresholds where appropriate	High	Strong
	B2	Pair BMI with waist circumference or WtHR for risk stratification	High	Strong
	B3	Primary care should measure BMI and waist circumference/WtHR annually	Moderate	Strong
	B4	Document obesity staging using ICD code E66	Moderate	Strong
	B5	Screen all newly diagnosed patients for metabolic, mechanical, hepatic, respiratory, reproductive, and psychological comorbidities	Moderate	Conditional
	B6	Baseline evaluation should include a core laboratory panel (glucose or HbA1c, lipid profile, ALT/AST, creatinine or eGFR, TSH), with additional tests performed when clinically indicated	Moderate	Strong
	B7	Perform sleep study when OSA is suspected; body composition/imaging for specialist care	Moderate	Strong
	B8	Prioritize screening in high-risk groups	Moderate	Strong
Treatment Principles	C1	Obesity management should include lifestyle therapy, psychological support, pharmacotherapy, and bariatric surgery	High	Strong
	C2	Primary goals: prevent progression, achieve remission when possible, reduce disease burden, improve quality of life	High	Strong
	C3	Treat obesity as a lifelong condition with long-term follow-up	High	Strong
	C4	Patients should be monitored regularly, typically every 3–6 months during active weight reduction or treatment initiation, with longer intervals (6–12 months) during stable phases, individualized according to disease severity, comorbidities, and care setting	Moderate	Strong
Pharmacotherapy	D1	OM indications: BMI ≥30; ≥27 with comorbidity; or ≥25 with WtHR ≥0.5 plus complication	High	Strong
	D2	Use stage-based treatment: Stage 0–1 = lifestyle ± OM; Stage 2–3 = intensive OM ± bariatric surgery	Moderate	Strong
	D3	First-line OMs: tirzepatide, semaglutide 2.4 mg; liraglutide as alternative; orlistat for cost-driven cases	High	Strong
​	D4	If <5% weight reduction is achieved after 6 months on a maximally tolerated dose, adherence and secondary causes should be reassessed, followed by modification of the treatment strategy, most commonly by switching therapy	High	Strong
	D5	Match pharmacotherapy to dominant comorbidity (T2D, MASLD, CVD, HF, OSA, OA)	Moderate	Strong
	D6	Selection of pharmacotherapy should be primarily based on clinical appropriateness, while also considering cost-effectiveness and real-world access to ensure equitable care	Moderate	Conditional
Bariatric/Metabolic Surgery	E1	Indication: BMI ≥40 or ≥35 with comorbidity	High	Strong
	E2	Metabolic/bariatric surgery may be considered in highly selected patients with uncontrolled type 2 diabetes and BMI ≥30 kg/m^2^, after failure or intolerance of optimal pharmacological and lifestyle therapy, following multidisciplinary evaluation	Moderate	Conditional
	E3	Requires multidisciplinary evaluation and lifelong follow-up	High	Strong
Health System and Policy	F1	Croatia should classify obesity as a chronic disease within national healthcare system	Moderate	Strong
	F2	Reimbursement should include modern OMs	Moderate	Strong
	F3	Develop regional obesity centres of excellence	Low–Moderate	Strong
	F4	Implement publicly funded lifestyle programs in primary care	Moderate	Strong
	F5	Provide national training programs to address stigma and improve obesity care	Moderate	Strong

### 3.2 Evidence grading and strength of recommendations

The quality of evidence and strength of recommendations were graded using a framework adapted from the GRADE methodology and recent European consensus documents (Guyatt et al., 2008). Evidence quality was classified as high, moderate, or low based on study design, consistency, and clinical relevance, while recommendation strength (strong or conditional) reflected the balance of benefits and risks, expert agreement, feasibility, and applicability within the Croatian healthcare system. Strong recommendations indicate high confidence in net clinical benefit and broad applicability, whereas conditional recommendations support individualized decision-making in settings with greater uncertainty or resource constraints.

## 4 Definition and diagnosis

### 4.1 Conceptual definition

Obesity is a chronic, progressive, relapsing, systemic disease characterized by excess or dysfunctional adipose tissue that impairs health (Busetto et al., 2024; Rubino et al., 2025; De Luca et al., 2024; Grunvald et al., 2022). Contemporary international frameworks emphasize that obesity cannot be defined by body size alone; rather, BMI serves as a screening tool and not a diagnostic endpoint (Busetto et al., 2024; Guidelines, 2025; Rubino et al., 2025). Diagnosis requires confirmation of increased adiposity together with objective evidence of health risk or dysfunction, such as metabolic, mechanical, or psychological consequences (Busetto et al., 2024; Guidelines, 2025; Rubino et al., 2025; Grunvald et al., 2022). This conceptualization aligns obesity with other long-term noncommunicable diseases and underscores the need for early identification and sustained management.

### 4.2 Diagnosis and classification of obesity

Diagnosis of obesity integrates anthropometric measures with clinical evidence of dysfunction, reflecting a shift from weight-based criteria toward a disease-based framework consistent with Lancet 2025 and EASO 2025 recommendations (Rubino et al., 2025).

### 4.3 Anthropometric component

Anthropometric assessment remains the first step in identifying individuals at risk. BMI categories follow WHO standards: normal weight (18.5–24.9 kg/m^2^), overweight (25–29.9 kg/m^2^), Class I obesity (30–34.9 kg/m^2^), Class II obesity (35–39.9 kg/m^2^), and Class III obesity (≥40 kg/m^2^) (Busetto et al., 2024; Guidelines, 2025; Rubino et al., 2025).

In the Croatian context, BMI thresholds are applied broadly, while lower cut-offs are recommended for individuals of Asian origin due to differences in adiposity-related risk (overweight ≥23 kg/m^2^, obesity ≥27.5 kg/m^2^) (Busetto et al., 2024; Guidelines, 2025; Rubino et al., 2025).

Waist-to-height ratio (WtHR) provides additional risk stratification and is now endorsed by NICE 2025 (Guidelines, 2025) as a key indicator of cardiometabolic risk, with thresholds of <0.5 (healthy), 0.5–0.59 (increased risk), and ≥0.6 (high risk). In older adults, BMI alone may underestimate adiposity and cardiometabolic risk because of age-related changes in body composition, including sarcopenia and fat redistribution toward visceral depots; therefore, assessment of waist-to-height ratio and body composition is particularly important in this population (Busetto et al., 2024; Guidelines, 2025; Rubino et al., 2025; De Luca et al., 2024).

Waist circumference further refines risk assessment, with health-risk thresholds of >94 cm (increased risk) and >102 cm (high risk) in men, and >80 cm and >88 cm in women, respectively (Busetto et al., 2024; Guidelines, 2025; Rubino et al., 2025).

These measures identify individuals with excessive adiposity, but classification as a disease requires corresponding evidence of health impairment.

### 4.4 Clinical component

The clinical diagnosis of obesity is established when excess adiposity leads to measurable health consequences. This requires evaluation across the metabolic, mechanical, psychological, and functional domains, including insulin resistance, type 2 diabetes, dyslipidemia, hypertension, obstructive sleep apnea, osteoarthritis, depressive symptoms, eating disorders, mobility limitations, and sarcopenic obesity (Busetto et al., 2024; Guidelines, 2025; Rubino et al., 2025; De Luca et al., 2024; Grunvald et al., 2022).

The clinical component ensures that the diagnosis reflects actual disease burden rather than body size alone and supports individualized treatment planning. This multidimensional evaluation is especially relevant in older adults, in whom obesity frequently presents with sarcopenic obesity, frailty, osteoarthritis, and functional limitations rather than isolated metabolic abnormalities.

In older adults, additional phenotypes such as dynapenic obesity, osteosarcopenic obesity, and coexisting osteopenia or osteoporosis should be actively assessed. Evaluation should prioritize muscle function (e.g., grip strength, gait speed) over muscle mass alone and include assessment of bone mineral density (DEXA) where clinically indicated (Manini and Clark, 2012; Ilich et al., 2016; Cruz-Jentoft et al., 2019).

### 4.5 Lancet 2025 framework

The 2025 Lancet Commission proposes a two-tier diagnostic framework that distinguishes between preclinical and clinical obesity (Rubino et al., 2025):

- Preclinical Obesity: characterized by confirmed excess adiposity in the absence of end-organ dysfunction or functional limitation. Individuals remain asymptomatic but carry elevated risk for progression to clinical disease.
- Clinical Obesity: defined by the presence of obesity-related organ dysfunction or significant functional limitation associated with adiposity. Diagnostic criteria include:
  ∘ At least one measurable clinical sign of tissue or organ impairment (e.g., T2DM, MASLD, OSA, hypertension, PCOS), or
  ∘ Significant limitations in daily functioning, including mobility restrictions or impaired self-care.

This framework reframes obesity as a disease long before severe complications develop, allowing earlier intervention and stratified treatment intensity.

### 4.6 Staging of obesity (EASO 2025 framework)

The 2025 EASO staging system provides a structured approach to assessing disease severity by integrating adiposity measures with the presence and extent of obesity-related complications. This framework categorizes individuals into four stages based on clinical burden and functional impact (Busetto et al., 2024).

- Stage 0 represents increased adiposity without identifiable complications.
- Stage 1 includes individuals with early or mild obesity-related abnormalities such as prediabetes or borderline hypertension.
- Stage 2 reflects established disease, including type 2 diabetes, cardiovascular disease, or MASLD.
- Stage 3 denotes severe end-organ damage or significant disability, such as advanced heart failure or marked osteoarthritis limiting daily activities.

This staging system directly informs therapeutic decision-making. Individuals in Stages 0–1 are candidates for lifestyle intervention with optional pharmacotherapy to prevent disease progression. In contrast, those in Stages 2–3 require intensive medical therapy, often including high-potency obesity pharmacotherapy and, when appropriate, bariatric surgery (Busetto et al., 2024). The framework ensures that treatment intensity corresponds to disease severity, supporting a precision-medicine approach to obesity care.

### 4.7 NICE 2025 practical guidance framework

The 2025 NICE guideline emphasizes a comprehensive diagnostic strategy that integrates anthropometric measures with clinical evaluation to avoid under-recognition of complications in individuals whose BMI may not fully reflect adiposity-related risk (Guidelines, 2025). NICE endorses combining BMI with WtHR for all adults, as WtHR better predicts cardiometabolic risk and should be routinely used in primary care.

A key principle of the guideline is the avoidance of diagnostic overshadowing, ensuring that symptoms such as fatigue, joint pain, depression, or sleep disturbances are not automatically attributed to obesity without proper assessment. NICE further recommends systematic screening for metabolic, mechanical, hepatic, respiratory, reproductive, and psychological comorbidities at the time of diagnosis, regardless of BMI category.

This approach supports earlier detection of clinically significant disease and aligns with modern chronic disease models that emphasize prevention, risk modification, and individualized intervention.

### 4.8 Croatian adaptation

To operationalize international standards within Croatia’s healthcare system, a national diagnostic and management framework must account for local epidemiology, clinical capacity, and reimbursement policies. Primary care serves as the cornerstone of obesity detection, with annual measurement of BMI, waist circumference, and preferably waist-to-height ratio recommended for all adults. Electronic medical records should incorporate obesity staging (ICD E66) to ensure longitudinal monitoring and enable structured care pathways. Screening priority should be given to individuals at elevated risk, including those with a family history of T2DM or cardiovascular disease, postmenopausal women, pregnant and postpartum women, patients with psychiatric disorders, and individuals using weight-promoting medications. Diagnostic confirmation in Croatia should include anthropometric assessment along with a standardized laboratory panel (glucose/HbA1c, lipid profile, ALT/AST, creatinine, eGFR, TSH). Additional investigations—such as sleep studies for suspected obstructive sleep apnoea and imaging modalities including liver ultrasound, DEXA, or bioimpedance analysis—should be performed in specialized settings when clinically indicated. This structured diagnostic approach ensures accurate disease classification and supports timely initiation of lifestyle, pharmacological, and surgical interventions. Furthermore, it is particularly valuable in aging populations because it incorporates functional impairment and organ damage, allowing treatment intensity to be matched to biological rather than chronological age (Table 2).

**TABLE 2:** Staging of obesity determines treatment focus.

Stage	Criteria	Treatment focus
Stage 0 – Preclinical Obesity	BMI ≥30 kg/m^2^, no current organ or functional impairment	Lifestyle modification; annual monitoring; consider preventive pharmacotherapy (depending on patients’ preferences)
Stage 1 – Mild Obesity	Mild functional impact (e.g., elevated blood pressure, insulin resistance)	Structured lifestyle program + pharmacotherapy (consider incretin-based therapy); referral to dietitian and physiotherapist
Stage 2 – Moderate Obesity	Confirmed obesity-related disease (e.g., T2D, MASLD, PCOS, OSA) OR functional limitation	Intensive medical therapy (consider incretin-based therapy depending on evidence of organ protection); consider early referral to metabolic/bariatric surgery consult; psychological support
Stage 3 – Severe Obesity	Multiple comorbidities with end organ damage OR significant functional impairment (e.g., inability to perform ADLs, severe OSA, CHF)	Prioritize for surgical evaluation; combined pharmacologic (consider incretin-based therapy depending on evidence of organ protection) + surgical + multidisciplinary care; structured rehabilitation

ADLs, Activities of daily living; BMI, Body mass index; CHF, Chronic heart failure; MASLD, Metabolic dysfunction-associated steatotic liver disease; OSA, Obstructive sleep apnea; PCOS, Polycystic ovary syndrome; T2D, Type 2 diabetes.

Summary of Croatian adaptation:

- Primary care is the frontline: every adult should have BMI and waist circumference measured at least once a year
- Electronic medical records should include obesity staging as a chronic diagnosis (ICD code E66)
- Screening priorities: adults with family history of T2D/CVD, post-menopause, pregnancy follow-up, patients with psychiatric conditions, or long-term medication use that increases weight (Busetto et al., 2024; Guidelines, 2025; Rubino et al., 2025; De Luca et al., 2024; Grunvald et al., 2022).
- Diagnostic confirmation in Croatia should include (Busetto et al., 2024; Guidelines, 2025; Rubino et al., 2025; De Luca et al., 2024; Grunvald et al., 2022):
o BMI + waist circumference or WtHR (preferable).
o Lab workup: glucose/HbA1c, lipid profile, ALT/AST, creatinine, TSH.
o Sleep study if OSA suspected.
o Imaging (liver ultrasound, DEXA, BIA) in specialized centres when needed (Busetto et al., 2024; Guidelines, 2025; Rubino et al., 2025; De Luca et al., 2024; Grunvald et al., 2022; McGowan et al., 2025).

## 5 Principles of management

Obesity management requires a chronic disease framework that integrates lifestyle strategies, psychological support, pharmacotherapy, and metabolic surgery according to disease stage, comorbidity profile, and patient preference (Busetto et al., 2024; Guidelines, 2025; Rubino et al., 2025; De Luca et al., 2024; Grunvald et al., 2022; McGowan et al., 2025; World Health Organization, 2025). The overarching goals of treatment are to prevent progression from preclinical to clinical obesity, achieve remission or improvement of complications, reduce cardiometabolic and mechanical burden, and enhance quality of life and psychological wellbeing.

### 5.1 Treatment goals

Because obesity is a relapsing chronic disease, treatment aims extend beyond weight reduction alone. Key objectives include improving metabolic health, preventing or delaying development of obesity-related diseases, supporting remission where possible (e.g., prediabetes or MASLD), and reducing functional limitations such as those caused by osteoarthritis or obstructive sleep apnoea (Busetto et al., 2024; Guidelines, 2025; Rubino et al., 2025; De Luca et al., 2024; Grunvald et al., 2022; McGowan et al., 2025; World Health Organization, 2025). Treatment goals should be individualized and reassessed regularly, recognizing that modest and sustained weight reduction may confer disproportionate health benefits in many patients. In older adults, treatment goals should prioritize preservation of muscle mass, muscle function, bone mass (Ilich et al., 2016; Cruz-Jentoft et al., 2019) alongside cardiometabolic risk reduction, rather than focusing exclusively on absolute weight reduction.

### 5.2 Core pillars of therapy

Obesity care encompasses four interdependent pillars:

#### 5.2.1 Lifestyle therapy

Foundational to all treatment plans, lifestyle intervention includes tailored medical nutrition therapy, structured physical activity, sleep optimization, and stress management. Multidisciplinary support—particularly dietitians and physiotherapists—is recommended when available (Busetto et al., 2024; Guidelines, 2025; Rubino et al., 2025; De Luca et al., 2024; Grunvald et al., 2022; McGowan et al., 2025; World Health Organization, 2025; Wadden et al., 2023). High-quality evidence demonstrates that structured lifestyle interventions combining dietary modification, physical activity, and behavioral support produce clinically meaningful weight reduction and improve cardiometabolic outcomes, particularly when delivered within multidisciplinary programs (Wing et al., 2013; Liechti et al., 2025; Gregori et al., 2025; Knowler et al., 2002; Lean et al., 2018; Appel et al., 2011; Romero-Gómez et al., 2017).

#### 5.2.2 Psychological Support

Behavioral and psychological interventions, including cognitive behavioral therapy and structured behavioral programs, significantly improve adherence, weight maintenance, and long-term outcomes, particularly when integrated with medical therapy (Hartmann-Boyce et al., 2014; Kurnik Mesarič et al., 2023; Gostoli et al., 2024; Dombrowski et al., 2014; Fahami et al., 2025).

Access to psychological services remains limited in many settings, but their inclusion improves adherence and long-term outcomes (Busetto et al., 2024; Guidelines, 2025; Rubino et al., 2025; De Luca et al., 2024; Grunvald et al., 2022).

#### 5.2.3 Pharmacotherapy

Obesity medications (OMs) serve as evidence-based adjuncts to lifestyle therapy and are indicated for adults with BMI ≥30 kg/m^2^ or ≥27 kg/m^2^ with weight-related complications, with optional extension to BMI ≥25 kg/m^2^ when waist-to-height ratio and clinical features indicate elevated cardiometabolic risk (Busetto et al., 2024; Guidelines, 2025; Rubino et al., 2025; De Luca et al., 2024; Grunvald et al., 2022; McGowan et al., 2025; World Health Organization, 2025).

#### 5.2.4 Metabolic/Bariatric Surgery

An option for individuals with severe obesity or obesity complicated by uncontrolled metabolic disease, surgery remains the most effective intervention for long-term weight reduction and disease modification in selected patients (Busetto et al., 2024; Guidelines, 2025; Rubino et al., 2025; Grunvald et al., 2022).

### 5.3 Special considerations in older adults

Obesity management in older adults requires individualized assessment of functional status, sarcopenia risk, multimorbidity burden, polypharmacy, and life expectancy. Treatment intensity should be guided by clinical stage, functional reserve, and patient priorities rather than age alone. Moderate weight reduction combined with resistance exercise and adequate protein intake is often preferable to aggressive weight reduction, with emphasis on preservation of fat-free mass, muscle function, bone health that are central to maintaining independence and healthy longevity (Di Vincenzo et al., 2025). Assessment of sarcopenic obesity in clinical practice should include evaluation of body composition (DEXA or bioelectrical impedance analysis where available), muscle strength (handgrip dynamometry), and physical performance (gait speed or chair stand test). Monitoring is recommended at baseline and at 6–12 months intervals in patients undergoing weight reduction therapy. Particular caution is required in individuals with rapid weight loss or multimorbidity, where preservation of lean mass should guide therapeutic intensity (Cruz-Jentoft et al., 2019; Prado et al., 2012; Donini et al., 2022).

### 5.4 Pharmacotherapy

Pharmacotherapy is a cornerstone of modern obesity management and is recommended as part of a comprehensive, chronic disease approach. Medications should be initiated based on BMI thresholds, disease stage, comorbidity profile, and patient-specific priorities. While traditional BMI thresholds guide eligibility, pharmacotherapy may also be considered based on adiposity-related complications and overall clinical risk, consistent with evolving international regulatory frameworks. All OMs should be prescribed alongside structured lifestyle therapy, with regular reassessment of efficacy and tolerability (Busetto et al., 2024; Guidelines, 2025; Rubino et al., 2025; De Luca et al., 2024; Grunvald et al., 2022; McGowan et al., 2025; World Health Organization, 2025).

General Indications.

- BMI ≥30 kg/m^2^, or
- BMI ≥27 kg/m^2^ with at least one obesity-related complication (e.g., prediabetes, hypertension, dyslipidaemia, OSA, MASLD), or
- BMI ≥25 kg/m^2^ with increased cardiometabolic risk indicated by WtHR ≥0.5 (Busetto et al., 2024; Guidelines, 2025; Rubino et al., 2025; De Luca et al., 2024; Grunvald et al., 2022; McGowan et al., 2025; World Health Organization, 2025).

Pharmacotherapy should be continued long term to maintain weight reduction and cardiometabolic benefits, similar to pharmacological therapy for hypertension or diabetes.

### 5.5 Obesity medications available in Croatia

A range of obesity medications is available in Croatia, differing in potency, mechanism of action, tolerability, and metabolic effects (Table 3). Selection should be individualized based on treatment goals, expected weight-loss efficacy, metabolic profile, and patient preference.

**TABLE 3:** Obesity medications: Efficacy, titration, and side effects.

Medication	Mean weight reduction (%)	Titration	Key side effects	Grade
Tirzepatide 5–15 mg	15%–21% (Jastreboff et al., 2022; Wang et al., 2024; Jastreboff et al., 2025)	Start 2.5 mg weekly → increase q4w to 10–15 mg	GI intolerance, gallbladder risk (Jastreboff et al., 2022; Wang et al., 2024; Jastreboff et al., 2025)	High; strong (Grunvald et al., 2022; Jastreboff et al., 2022; Wang et al., 2024; Wilding et al., 2021)
Semaglutide 2.4–7.2 mg	12%–15% (Rubino et al., 2021; Perreault et al., 2022; Wilding et al., 2021; Wharton et al., 2025a; Pi-Sunyer et al., 2015)	Start 0.25 mg weekly → increase q4w to 2.4 mg	GI intolerance, gallbladder risk (Grunvald et al., 2022; Rubino et al., 2021; Perreault et al., 2022; Wharton et al., 2025a)	High; strong (Grunvald et al., 2022; Rubino et al., 2021; Perreault et al., 2022; Wilding et al., 2021; Wharton et al., 2025a)
Liraglutide 3.0 mg	7%–9% (Pi-Sunyer et al., 2015; Davies et al., 2015; le Roux et al., 2017)	Start 0.6 mg daily → increase weekly to 3.0 mg	Nausea, vomiting, injection burden (Grunvald et al., 2022; Pi-Sunyer et al., 2015; Davies et al., 2015)	High; strong (Grunvald et al., 2022; Wilding et al., 2021; Pi-Sunyer et al., 2015; Davies et al., 2015)
Naltrexone/Bupropion	5%–6% (Apovian et al., 2013; Nissen et al., 2016)	Titrate to 2 tabs BID over 4 weeks	Nausea, insomnia, ↑ BP (Grunvald et al., 2022; Apovian et al., 2013; Nissen et al., 2016)	Moderate; conditional (Grunvald et al., 2022; Apovian et al., 2013; Nissen et al., 2016)
Orlistat	3%–5% (Finer et al., 2000; Torgerson et al., 2004)	120 mg TID with meals	GI (steatorrhea, diarrhea) (Finer et al., 2000; Torgerson et al., 2004; Sahebkar et al., 2018)	Moderate; conditional (Finer et al., 2000; Torgerson et al., 2004; Sahebkar et al., 2018)

BID, Twice daily; BP, Blood pressure; GI, Gastrointestinal; q4w—Every 4 weeks; TID, Three times daily.

#### 5.5.1 Tirzepatide 5–15 mg (dual GIP/GLP-1 receptor agonist)

Tirzepatide is the most effective medication for weight reduction currently available, achieving average losses of 15%–21% across clinical trials (Wadden et al., 2023; Jastreboff et al., 2022; Wang et al., 2024; Jastreboff et al., 2025). It produces large reductions in HbA1c and a marked decrease in progression from prediabetes to type 2 diabetes. Modest reductions in systolic blood pressure and improvements in triglycerides and non-HDL cholesterol are consistently observed. Sustained weight reduction was demonstrated in the SURMOUNT-4 trial, supporting the role of long-term pharmacotherapy as a component of chronic obesity management (Jastreboff et al., 2022).

Gastrointestinal side effects are the most common, typically during dose escalation. Because of its high potency and strong metabolic effects, tirzepatide is appropriate for individuals seeking substantial weight reduction with additional improvements in glycemic and lipid parameters.

#### 5.5.2 Semaglutide 2.4–7.2 mg (GLP-1 receptor agonist)

Semaglutide results in 12%–15% weight reduction in the STEP trials (Wang et al., 2024; Rubino et al., 2021; Perreault et al., 2022; Wilding et al., 2021) and produces clinically meaningful improvements in glycaemia, including regression from prediabetes. It also modestly lowers systolic and diastolic blood pressure and improves triglyceride and cholesterol fractions. Maintenance of weight reduction has been demonstrated in STEP-4 trial, supporting long-term pharmacotherapy as a chronic treatment strategy (Rubino et al., 2021). Recent data from the STEP UP trial demonstrate that a higher investigational dose of semaglutide 7.2 mg once weekly leads to even greater weight reduction, with mean weight reduction of approximately 20%–21% at 72 weeks, significantly exceeding the effect observed with the 2.4 mg dose. Gastrointestinal symptoms are dose-related and generally manageable with gradual titration. Semaglutide is suited for individuals prioritizing significant weight reduction with consistent improvements in multiple cardiometabolic risk factors. Semaglutide 7.2 mg is not available in Croatia (Wharton et al., 2025a).

#### 5.5.3 Liraglutide 3.0 mg (GLP-1 receptor agonist)

Liraglutide produces moderate weight reduction of 7%–9% (Pi-Sunyer et al., 2015; Davies et al., 2015; le Roux et al., 2017) and improves glycemic control with demonstrated reductions in progression from prediabetes to diabetes. Blood pressure improvements are modest, and lipid effects include reductions in triglycerides and LDL cholesterol. Daily injections and gastrointestinal symptoms may limit long-term adherence in some patients, but liraglutide remains an effective option when weekly incretin therapies are unavailable or not preferred.

#### 5.5.4 Naltrexone/bupropion (oral combination)

Naltrexone/bupropion leads to weight reduction of approximately 5%–6% (le Roux et al., 2017) and offers small improvements in HDL cholesterol and triglycerides. However, it may increase blood pressure and heart rate and is contraindicated in patients with uncontrolled hypertension (le Roux et al., 2017; Apovian et al., 2013). It is an alternative for individuals unable to use incretin-based therapies or those preferring an oral medication; however, it is not available in Croatia.

#### 5.5.5 Orlistat

Orlistat provides modest weight reduction of 3%–5% but has favorable effects on LDL cholesterol, total cholesterol, and blood pressure, supported by multiple randomized trials (Finer et al., 2000; Torgerson et al., 2004; Nissen et al., 2016; Sahebkar et al., 2017). Gastrointestinal side effects may limit adherence, though they also reinforce dietary fat restriction. Its major advantage is full reimbursement in Croatia, making it the most accessible pharmacological option in low-resource settings.

#### 5.5.6 Emerging therapies

Emerging therapies include oral semaglutide and non-peptide incretin agonists such as orforglipron, which may further expand therapeutic options (Wharton et al., 2023; Wharton et al., 2025b).

### 5.6 Stratification of patients

#### 5.6.1 Stage-based stratification

Pharmacological recommendations in this position statement are aligned with the European Association for the Study of Obesity framework and are designed to support individualized, stage-based treatment across the lifespan, including older adults with multimorbidity or functional impairment (Di Vincenzo et al., 2025; McGowan et al., 2025). This approach is consistent with the World Health Organization (WHO) guideline on the use of incretin-based therapies for obesity in adults, which conditionally endorses long-term use of GLP-1 and dual GIP/GLP-1 receptor agonists as part of an integrated, chronic disease management model, with explicit consideration of equity, access, and health-system capacity (World Health Organization, 2025).

Therefore, treatment should be tailored based on disease stage and presence of complications:

- Preclinical Obesity: Focus on weight-loss efficacy and prevention of disease progression. High-potency agents (tirzepatide, semaglutide) are appropriate for motivated individuals or those preparing for orthopedic or reproductive interventions.
- Clinical Obesity: Therapy should match the dominant comorbidity—prioritizing cardiovascular, hepatic, respiratory, metabolic, or musculoskeletal benefits as indicated.

### 5.7 RCT evidence: obesity pharmacotherapy and prevention/treatment of comorbidities

#### 5.7.1 Effects on cardiometabolic risk factors of obesity medications

Obesity medications provide clinically meaningful improvements across multiple cardiometabolic risk factors beyond weight reduction. GLP-1 and GIP/GLP-1 receptor agonists demonstrate the strongest effects (Table 4). Semaglutide improves glycemic control, enhances regression from prediabetes, and reduces progression to type 2 diabetes, as shown consistently in the STEP trials (Rubino et al., 2021; Perreault et al., 2022; Wilding et al., 2021; Wharton et al., 2025a; Salamah et al., 2024), while tirzepatide achieves even greater glycemic reductions and an approximately 90% reduction in incident diabetes in the SURMOUNT-1 extension (Wadden et al., 2023; Jastreboff et al., 2022; Jastreboff et al., 2025; Salamah et al., 2024). Semaglutide also reduced major adverse cardiovascular events by 20% in SELECT (Lincoff et al., 2023), whereas tirzepatide reduced heart-failure hospitalizations and composite cardiovascular events in SUMMIT (Packer et al., 2025). Semaglutide significantly improved symptoms and functional capacity in HFpEF populations in STEP-HFpEF (Kosiborod et al., 2024).

**TABLE 4:** Effects of obesity medications on cardiometabolic risk factors.

Medication (with key references)	Glycaemic effects	Blood pressure	Lipid effects	Grade
Tirzepatide 5–15 mg (Wadden et al., 2023; Jastreboff et al., 2022; Jastreboff et al., 2025; Salamah et al., 2024; Liu et al., 2024; Krumholz et al., 2024)	Large HbA1c reduction; strong diabetes prevention	↓ SBP (modest)	↓ TG, ↓ non-HDL; ↑ HDL	High/Strong
Semaglutide 2.4–7.2 mg (Rubino et al., 2021; Perreault et al., 2022; Wilding et al., 2021; Wharton et al., 2025a; Pi-Sunyer et al., 2015; Salamah et al., 2024; Liu et al., 2024)	Significant HbA1c improvement; T2D prevention	↓ SBP/DBP	Improved TG, LDL, non-HDL	High/Strong
Liraglutide 3.0 mg (Pi-Sunyer et al., 2015; Davies et al., 2015; le Roux et al., 2017; Salamah et al., 2024; Liu et al., 2024)	HbA1c improvement; 79% ↓ progression to diabetes	↓ SBP (small)	Improved TG and LDL	High/Strong
Naltrexone/Bupropion (Apovian et al., 2013; Nissen et al., 2016)	Small HbA1c improvement	Neutral or slight ↑ BP/HR	Mild HDL ↑, modest TG ↓	Moderate/Conditional
Orlistat (Finer et al., 2000; Torgerson et al., 2004; Sahebkar et al., 2017; Sahebkar et al., 2018)	Small glycaemic benefit; ↓ T2D incidence	↓ SBP/DBP (small)	↓ LDL, ↓ total cholesterol; modest ↑ HDL	Moderate/Conditional

BP, Blood pressure; DBP, Diastolic blood pressure; HbA1c—Glycated hemoglobin; HDL, High-density lipoprotein cholesterol; HR, Heart rate; LDL, Low-density lipoprotein cholesterol; SBP, Systolic blood pressure; T2D, Type 2 diabetes; TG, Triglycerides.

In terms of blood pressure and lipid profiles, liraglutide and semaglutide produce modest reductions in systolic blood pressure (Wang et al., 2024; Rubino et al., 2021; Wilding et al., 2021; Wharton et al., 2025a; Pi-Sunyer et al., 2015; Davies et al., 2015; le Roux et al., 2017; Liu et al., 2024) and meaningful improvements in triglycerides and non-HDL cholesterol (Wang et al., 2024; Rubino et al., 2021; Wilding et al., 2021; Wharton et al., 2025a; Pi-Sunyer et al., 2015; Davies et al., 2015; le Roux et al., 2017; Liu et al., 2024). Orlistat reduces LDL-cholesterol and total cholesterol and leads to small decreases in blood pressure, supported by multiple trials (Finer et al., 2000; Torgerson et al., 2004; Sahebkar et al., 2017; Sahebkar et al., 2018). Naltrexone/bupropion offers modest improvements in triglycerides and HDL-cholesterol but may increase blood pressure and heart rate, as demonstrated in COR-I and CV safety analyses (Apovian et al., 2013; Nissen et al., 2016). Tirzepatide and semaglutide also show benefits in other obesity-related cardiometabolic conditions, including clinically significant reductions in apnea–hypopnea index in OSA (Malhotra et al., 2024; Li et al., 2025; Blackman et al., 2016) and improvements in osteoarthritis pain and function with semaglutide (Bliddal et al., 2024). Collectively, these effects support the preferential use of high-potency incretin-based therapies when accessible (Table 5).

**TABLE 5:** Summary of RCTs demonstrating benefits in preventing/improving obesity comorbidities.

Comorbidity/Clinical domain	Medication(s) (with references)	Key findings	Grade
Prediabetes Regression and Diabetes Prevention	Tirzepatide (Jastreboff et al., 2022; Jastreboff et al., 2025)	∼90% reduction in progression to T2D (SURMOUNT-1 extension)	High/Strong
	Semaglutide 2.4 mg (Rubino et al., 2021; Perreault et al., 2022; Wilding et al., 2021)	High reversion to normoglycaemia; reduced T2D incidence	High/Strong
	Liraglutide 3.0 mg (Pi-Sunyer et al., 2015; Davies et al., 2015; le Roux et al., 2017)	79% reduction in progression to diabetes (SCALE)	High/Strong
	Orlistat (Finer et al., 2000; Torgerson et al., 2004)	Reduced incidence of diabetes (XENDOS)	Moderate
MASLD/MASH	Semaglutide 2.4 mg (Sanyal et al., 2025)	Histologic MASH resolution + fibrosis improvement (ESSENCE)	High/Strong
	Tirzepatide (Loomba et al., 2024)	Resolution + fibrosis improvement (SYNERGY-MASH)	High/Strong
	Liraglutide 3.0 mg (Armstrong et al., 2016)	Early histologic improvement (LEAN)	Moderate
Cardiovascular Disease (ASCVD/MACE)	Semaglutide 2.4 mg (Lincoff et al., 2023)	20% reduction in major adverse CV events (SELECT)	High/Strong
Heart Failure (HFpEF) – Clinical Events	Tirzepatide (Packer et al., 2025)	↓ HF hospitalization and composite CV outcomes (SUMMIT)	High/Strong
Heart Failure (HFpEF) – Symptoms and Function	Semaglutide 2.4 mg (Kosiborod et al., 2024)	Improved KCCQ-CSS and 6-min walk distance (STEP-HFpEF)	Moderate (Symptom-Based)
Obstructive Sleep Apnea (OSA)	Tirzepatide (Malhotra et al., 2024; Li et al., 2025; Blackman et al., 2016)	Large AHI reductions; OSA remission; FDA-approved for OSA	High/Strong
	Liraglutide 3.0 mg (Li et al., 2025; Blackman et al., 2016)	Modest reduction in AHI (SCALE Sleep Apnea)	Moderate
Knee Osteoarthritis (OA)	Semaglutide 2.4 mg (Bliddal et al., 2024)	Clinically meaningful pain reduction + functional improvement	High/Strong

AHI, Apnea–hypopnea index; ASCVD, Atherosclerotic cardiovascular disease; CV, Cardiovascular; FDA—U.S., food and drug administration; HF, Heart failure; HFpEF, Heart failure with preserved ejection fraction; KCCQ-CSS, Kansas City Cardiomyopathy Questionnaire Clinical Summary Score; MACE, Major adverse cardiovascular events; MASLD, Metabolic dysfunction-associated steatotic liver disease; MASH, Metabolic dysfunction-associated steatohepatitis; NASH, Non-alcoholic steatohepatitis; OSA, Obstructive sleep apnea; OA, Osteoarthritis; T2D, Type 2 diabetes.

#### 5.7.2 Cardiovascular disease (CVD)

The SELECT trial (NEJM 2023) demonstrated that semaglutide 2.4 mg reduced major adverse cardiovascular events (MACE: CV death, nonfatal MI, nonfatal stroke) by 20% in people with overweight/obesity and established CVD but no diabetes (Lincoff et al., 2023). This is the first definitive RCT proving cardiovascular event prevention with an obesity agent. In addition, prespecified analyses showed reduced progression to diabetes and more regression to normoglycemia. For Croatia, semaglutide thus holds priority in patients with obesity and established CVD.

#### 5.7.3 Heart failure (HF)

Two GLP-1/GIP agonists have shown benefits in heart failure with preserved ejection fraction (HFpEF) populations with obesity. STEP-HFpEF (NEJM 2023) showed that semaglutide 2.4 mg improved symptoms (KCCQ-CSS) and exercise tolerance (6-min walk) over 52 weeks (Kosiborod et al., 2024). The SUMMIT trial (NEJM 2025) demonstrated that tirzepatide not only improved symptoms but also reduced the composite of CV death or worsening HF (HR 0.62, 95% CI 0.41–0.95) (Packer et al., 2025). This establishes tirzepatide as the first obesity pharmacotherapy with HF event reduction.

#### 5.7.4 Obstructive sleep apnea (OSA)

The SURMOUNT-OSA program tested tirzepatide in patients with moderate-to-severe OSA and obesity, both with and without CPAP (Malhotra et al., 2024). It produced large reductions in apnea–hypopnea index (−20 and −23.8 events/hour vs. placebo). Liraglutide 3.0 mg (SCALE Sleep Apnea, Int J Obes 2016) also reduced AHI modestly (−6.1 events/h difference) (Blackman et al., 2016). Tirzepatide is now FDA-authorized for OSA treatment in adults with obesity and would be the preferred drug in Croatian patients with this comorbidity.

#### 5.7.5 Knee osteoarthritis (OA)

A landmark NEJM 2024 trial tested semaglutide 2.4 mg in knee OA patients with obesity. Semaglutide produced clinically meaningful reductions in WOMAC pain scores and improved physical function over 68 weeks (Bliddal et al., 2024). This trial demonstrates that weight reduction via semaglutide can directly improve musculoskeletal outcomes.

#### 5.7.6 Metabolic dysfunction-associated steatotic liver disease (MASLD/MASH)

Liraglutide (LEAN, Lancet 2016) was the first RCT to show histological NASH resolution in a substantial proportion (39% vs. 9%) (Armstrong et al., 2016). Semaglutide has shown powerful effects: a phase two trial (NEJM 2021) demonstrated NASH resolution without worsening fibrosis, and the ESSENCE phase three (NEJM 2025) confirmed significant improvements in both steatohepatitis and fibrosis (Sanyal et al., 2025). Tirzepatide (SYNERGY-NASH, NEJM 2024) also showed high rates of MASH resolution with fibrosis improvement (Loomba et al., 2024). Taken together, both tirzepatide and semaglutide can be prioritized for patients with obesity and MASLD.

#### 5.7.7 Type 2 diabetes (prevention)

The SCALE Obesity and Prediabetes trial showed that liraglutide 3.0 mg reduced progression to diabetes by 79% (HR 0.21) over 160 weeks (Pi-Sunyer et al., 2015). Tirzepatide (SURMOUNT-1 3-year extension, NEJM 2025) showed an even stronger preventive effect, reducing incident diabetes to 1.3% vs. 13.3% with placebo over 176 weeks (HR 0.07) (Jastreboff et al., 2025). The STEP (Semaglutide Treatment Effect in People with obesity) clinical trial program tested weekly semaglutide 2.4 mg in adults with overweight or obesity (Rubino et al., 2021; Perreault et al., 2022; Wilding et al., 2021). Several STEP trials included large proportions of participants with prediabetes at baseline. Across all trials, semaglutide significantly increased regression to normoglycemia and reduced progression to type 2 diabetes compared to placebo (Rubino et al., 2021; Perreault et al., 2022; Wilding et al., 2021; Sahebkar et al., 2018). STEP 1 (NEJM 2021): 84% reverted to normoglycemia vs. 48% with placebo at 68 weeks; 0.5% vs. 3% developed diabetes (Rubino et al., 2021). STEP 3 and STEP 4: Similar improvements in glycemic outcomes and prevention of diabetes onset. STEP 5 (NEJM 2022, 104 weeks): 79% reverted to normoglycemia vs. 37% with placebo; very low incidence of diabetes on semaglutide. Semaglutide also showed diabetes prevention signals in SELECT (Packer et al., 2025). Thus, tirzepatide and semaglutide are the most potent preventive options, with liraglutide also offering strong protection.

### 5.8 Prioritization framework according to Croatian Endocrine Society

**FIGURE 1:**
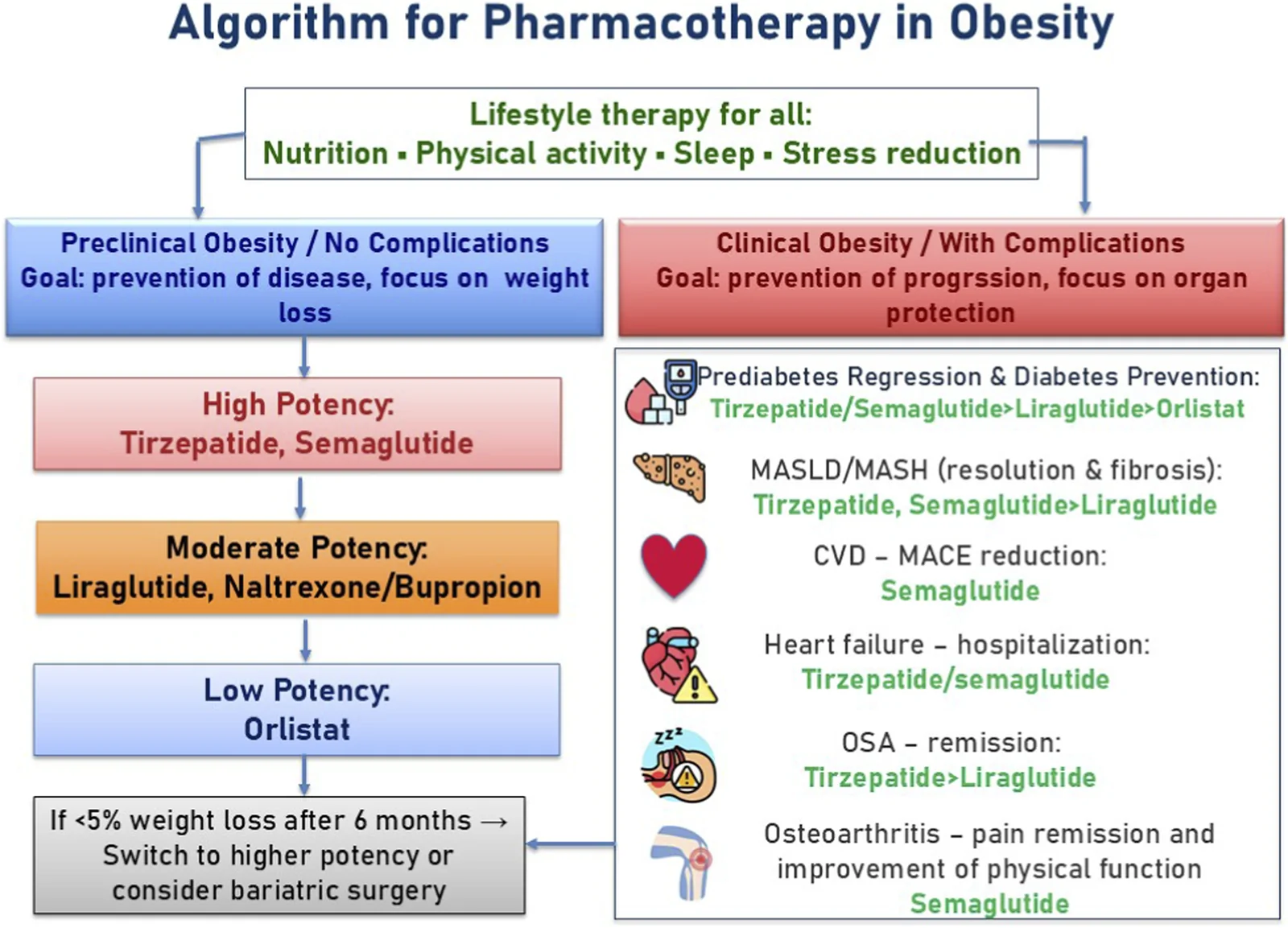
Algorithm for pharmacotherapy in obesity. Lifestyle intervention—including nutritional therapy, physical activity, adequate sleep, and stress reduction—represents the foundation of treatment for all patients. In preclinical obesity without established complications, pharmacotherapy is selected primarily according to expected weight-loss efficacy, progressing from high- to moderate- and low-potency options. In clinical obesity with obesity-related complications, treatment choice should be driven by evidence for complication-specific and organ-protective benefits, including prevention of type 2 diabetes, improvement of MASLD/MASH, reduction in major adverse cardiovascular events, improvement of heart failure outcomes, remission of obstructive sleep apnoea, and relief of osteoarthritis symptoms. Treatment response should be reassessed after approximately 6 months; insufficient weight loss should prompt escalation to a more effective therapy or consideration of metabolic–bariatric surgery.

The Croatian Endocrine Society prioritization framework provides a structured approach to selecting pharmacotherapy for obesity that integrates disease stage, dominant comorbidity profile, treatment goals, and real-world access considerations. This model translates contemporary international evidence into a pragmatic decision pathway adapted to national clinical practice and healthcare system constraints (Figure 1).

#### 5.8.1 Framework by disease stage

The CES prioritization framework emphasizes a stage-based and comorbidity-driven approach to pharmacotherapy, ensuring that treatment intensity matches disease severity (Table 6). In preclinical obesity, priority is given to preventing progression, with high-potency agents such as tirzepatide and semaglutide recommended when substantial weight reduction is desired, while moderate- and low-potency options remain appropriate when access or tolerance limits choices. In clinical obesity, therapy must be tailored to the dominant comorbidity profile, using agents with the strongest evidence for improving glycaemia, lipids, blood pressure, OSA severity, or musculoskeletal symptoms. Cost and access considerations are integral to CES guidance: orlistat remains the first-line reimbursed option in Croatia, while semaglutide, tirzepatide, and liraglutide are preferred when affordable due to their greater efficacy and metabolic benefits. This framework ensures equitable, evidence-based, and individualized treatment selection across diverse patient populations.

**TABLE 6:** Pharmacotherapy algorithm in obesity according to disease stage.

Clinical stage/Patient profile	Treatment goal	Preferred pharmacotherapy options	Notes	Grade
Preclinical Obesity Stage 0; no complications	Prevent onset of clinical obesity; weight reduction	High potency: Tirzepatide, Semaglutide *(High)* Moderate potency: Liraglutide *(High)* Low potency: Orlistat *(Moderate)*	If <5% weight reduction after 6 months, escalate potency or consider bariatric evaluation	High/Strong for high-potency incretins
Clinical Obesity Stages 1–3; complications present	Prevent progression; organ protection	Match OM to comorbidity • CVD/MACE: Semaglutide *(High)* • HFpEF: Tirzepatide or Semaglutide *(High)* • MASLD/MASH: Tirzepatide/Semaglutide *(High)* • Prediabetes/T2D: Tirzepatide/Semaglutide/Liraglutide *(High)* → Orlistat *(Moderate)* • OSA: Tirzepatide *(High)* > Liraglutide *(Moderate)* • OA: Semaglutide *(High)*	Comorbidity-driven therapy; refer Stage 2–3 for metabolic surgery evaluation	High/Strong for organ-protective incretins
Any Stage with inadequate response	Optimize long-term therapy	Switch to higher potency, change class, or combine with surgery	Reinforce lifestyle + behavioral therapy	High for escalation strategy

OM, obesity medication; CVD, Cardiovascular disease; HFpEF, Heart failure with preserved ejection fraction; MACE, Major adverse cardiovascular events; MASLD, Metabolic dysfunction-associated steatotic liver disease; MASH, Metabolic dysfunction-associated steatohepatitis; OA, Osteoarthritis; OSA, Obstructive sleep apnea; T2D, type 2 diabetes.

#### 5.8.2 Framework by cost/access

In the Croatian healthcare context, where reimbursement limitations significantly influence treatment availability, prioritization of obesity medications must balance clinical efficacy with affordability (Table 7). Orlistat remains the only widely reimbursed option and therefore serves as the first-line therapy in situations where cost is the primary barrier, despite its modest efficacy and limited metabolic impact. When patients have the financial means or supplemental coverage, higher-potency incretin-based therapies—semaglutide 2.4 mg and tirzepatide—should be prioritized given their superior weight-loss efficacy and strong evidence for organ protection, including benefits in cardiovascular disease, heart failure, MASLD/MASH, osteoarthritis, prediabetes regression, and obstructive sleep apnea. Liraglutide 3.0 mg occupies an intermediate tier, offering clinically meaningful weight reduction and preventive metabolic benefits at a lower cost than newer incretin agents, making it a suitable alternative when semaglutide or tirzepatide are inaccessible. Naltrexone/bupropion is not availble. This cost-sensitive hierarchy ensures equitable access while optimizing clinical outcomes whenever higher-efficacy therapies are feasible.

**TABLE 7:** Prioritization by cost and access (Croatian context).

Cost tier (Croatian context)	Medications	Weight reduction potency	Recommended use	Grade
First-Line (Reimbursed/Low Cost)	Orlistat	Low potency	When cost is limiting, modest metabolic benefit	Moderate/Conditional
Preferred when financially accessible	Semaglutide/Tirzepatide	High potency	Best for weight reduction and organ protection (CVD, HFpEF, MASLD, OSA, OA)	High/Strong
Alternative Tier	Liraglutide	Moderate potency	For intolerance, contraindications, or restricted access to high-potency agents	Moderate

CVD, Cardiovascular disease; HFpEF, Heart failure with preserved ejection fraction; MASLD, Metabolic dysfunction-associated steatotic liver disease; OA, Osteoarthritis; OSA, Obstructive sleep apnea.

### 5.9 Stopping rules for pharmacotherapy

Effective use of obesity pharmacotherapy requires structured reassessment to ensure that treatment remains beneficial, safe, and aligned with therapeutic goals. Because treatment response varies substantially, patients should be evaluated approximately 12 weeks after reaching the maximally tolerated dose and reassessed comprehensively at 3–6 months, depending on medication characteristics and individual factors (Busetto et al., 2024; Guidelines, 2025; Rubino et al., 2025; McGowan et al., 2025; World Health Organization, 2025).

Treatment modification should be considered when response is inadequate. A weight reduction of <5% after 3–6 months at full dose represents the minimum threshold indicating insufficient effect, even for high-potency agents such as dual GIP/GLP-1 or high-dose GLP-1 receptor agonists. In addition to weight change, clinicians should assess improvement in metabolic and clinical parameters, including glycaemia, blood pressure, lipid profile, hepatic markers, and symptom burden. Lack of improvement despite confirmed adherence suggests suboptimal therapeutic benefit.

Therapy should also be reconsidered if persistent adverse effects occur or if practical barriers—such as cost, regimen complexity, or injection intolerance—limit long-term use. When stopping criteria are met, timely adjustment is recommended, including switching to a more potent or better-tolerated agent, changing drug class, intensifying lifestyle intervention, or evaluating eligibility for metabolic-bariatric surgery in patients with advanced disease or ongoing complications.

Implementation of structured stopping rules supports rational pharmacotherapy, prevents prolonged ineffective treatment, and promotes timely escalation to interventions more likely to achieve clinically meaningful outcomes.

## 6 Bariatric (metabolic) surgery

Metabolic–bariatric surgery is the most effective evidence-based intervention for achieving substantial and durable weight reduction and improving obesity-related complications, and it should be considered part of a comprehensive chronic disease management strategy rather than a last-resort therapy (Busetto et al., 2024; Guidelines, 2025; Rubino et al., 2025; De Luca et al., 2024; Grunvald et al., 2022). Current indications follow international guideline recommendations and include BMI ≥40 kg/m^2^ regardless of comorbidities, BMI ≥35 kg/m^2^ with at least one significant obesity-related complication, and selected individuals with BMI ≥30 kg/m^2^ whose type 2 diabetes remains inadequately controlled despite optimal medical therapy (De Luca et al., 2024). Evidence increasingly supports the metabolic benefits of surgery in carefully selected patients at lower BMI thresholds, particularly for glycaemic control and cardiometabolic risk reduction when optimized pharmacotherapy has failed to achieve adequate disease control (De Luca et al., 2024).

Preoperative assessment should be multidisciplinary and performed in accredited centres, incorporating cardiometabolic evaluation, psychological and nutritional assessment, and screening for obesity-associated conditions such as obstructive sleep apnea and metabolic dysfunction-associated steatotic liver disease. Assessment should also include bone mineral density, particularly in older adults and those at risk of osteopenia or osteoporosis. The most commonly performed procedures—sleeve gastrectomy, Roux-en-Y gastric bypass, and one-anastomosis gastric bypass—consistently produce significant long-term weight reduction and improvement in metabolic disease (Busetto et al., 2024; Guidelines, 2025; Rubino et al., 2025; De Luca et al., 2024; Grunvald et al., 2022). Special consideration is required in older adults, in whom surgical decision-making should prioritize functional status, sarcopenia risk, comorbidity burden, and anticipated benefits for mobility, independence, and quality of life (Di Vincenzo et al., 2025).

Lifelong postoperative follow-up is essential and should include monitoring for micronutrient deficiencies, surveillance of metabolic and obesity-related conditions, and ongoing behavioral and nutritional support (Busetto et al., 2024; Guidelines, 2025; Rubino et al., 2025; De Luca et al., 2024; Grunvald et al., 2022). Because obesity is a chronic disease, adjunct pharmacotherapy may be required if weight regain or metabolic relapse occurs, supporting a longitudinal, integrated treatment model (De Luca et al., 2024; Grunvald et al., 2022).

## 7 Long-term follow-up

Given the chronic, relapsing nature of obesity, structured long-term follow-up is essential to maintain weight reduction, prevent recurrence of complications, and preserve functional capacity, particularly in older adults at increased risk of frailty, sarcopenia, and disability (Busetto et al., 2024; Guidelines, 2025; Rubino et al., 2025; De Luca et al., 2024; Grunvald et al., 2022). Follow-up intensity should be individualized according to treatment phase and disease complexity. During active weight reduction or pharmacotherapy titration, reassessment is generally recommended every 3–6 months, whereas patients in stable maintenance phases may be reviewed at 6–12-month intervals. More frequent monitoring is appropriate for individuals with complex comorbidities, including type 2 diabetes, heart failure with preserved ejection fraction, metabolic dysfunction-associated steatotic liver disease, or obstructive sleep apnea, in whom treatment adjustments and complication surveillance may be required.

Follow-up evaluations should incorporate multidimensional clinical assessment. Anthropometric monitoring should include weight, body mass index, waist circumference, and waist-to-height ratio to detect changes in adiposity and cardiometabolic risk. Metabolic and mechanical complications should be periodically reassessed through evaluation of glycaemia, lipid profile, liver function, blood pressure, sleep-related symptoms, and functional limitations such as osteoarthritis-related pain. Particular attention should be given to early detection of dynapenia and decline in muscle function. Medication review is also necessary to determine efficacy, tolerability, adherence, and whether predefined response thresholds—such as insufficient weight reduction after adequate treatment duration—indicate the need for therapy modification.

Sustained behavioral and nutritional support represents a core component of long-term management, as reinforcement of dietary, physical activity, sleep, and psychological strategies improves treatment adherence and durability of outcomes. Routine screening for depression, anxiety, emotional eating, and the effects of weight stigma is recommended because psychological factors strongly influence treatment response and relapse risk. Effective follow-up should also be multidisciplinary, involving coordinated collaboration between primary care providers and relevant specialists, including endocrinology, hepatology, cardiology, sleep medicine, orthopaedics, and bariatric surgery teams, to ensure comprehensive management of obesity and its complications.

Overall, structured longitudinal monitoring supports early detection of relapse or complications, enables timely treatment adjustment, and reinforces the chronic disease care model required for effective long-term obesity management.

## 8 Policy recommendations for Croatia

A comprehensive national strategy is required to address obesity as a chronic progressive disease. The following actions align with contemporary international guidelines (Busetto et al., 2024; Guidelines, 2025; Rubino et al., 2025; De Luca et al., 2024; Grunvald et al., 2022) and Croatia’s healthcare structure.

1. Formal Classification of Obesity as a Chronic Disease

Formal recognition of obesity as a chronic disease by the Croatian National Health Fund would enable structured care pathways.

2. Expansion of Reimbursement for Evidence-Based Pharmacotherapies

Reimbursement should extend beyond orlistat to include high-efficacy agents (tirzepatide, semaglutide 2.4 mg, liraglutide 3.0 mg), which provide superior weight-loss and metabolic improvements.

3. Establish Regional Centres of Excellence

Multidisciplinary obesity management centres should be developed to deliver integrated care, including dietary counselling, physiotherapy, psychological support, pharmacotherapy, and metabolic surgery.

4. Fund Structured Lifestyle and Behavioural Programs

Nationally funded, evidence-based lifestyle programs should be delivered in primary care and community settings to ensure early intervention and ongoing support.

5. Professional Training and Anti-Stigma Initiatives

Comprehensive education on obesity management and stigma reduction must be integrated into medical training and continuing professional development.

6. Routine Obesity Screening in Primary Care

BMI, waist circumference, and WtHR should be measured annually for all adults, with EMR prompts for obesity staging and comorbidity assessment.

7. National Surveillance and Research Infrastructure

A national obesity registry should be developed to monitor prevalence, outcomes, treatment uptake, and cost-effectiveness.

8. Support access to emerging therapies including oral semaglutide and non-peptide incretin therapies (e.g., orforglipron).

## 9 Discussion

This position statement synthesizes contemporary scientific evidence, international consensus frameworks, and national clinical realities into a unified model for obesity diagnosis and management. Its central premise is that obesity should be approached as a chronic, relapsing, systemic progressive disease rather than a risk factor defined solely by anthropometric thresholds (Busetto et al., 2024; Guidelines, 2025; Rubino et al., 2025). This paradigm shift reflects a growing global consensus that disease burden, functional impairment, and complication risk—not body size alone—should guide clinical decision-making.

A major strength of this statement is its integration of staging-based assessment with treatment selection. By aligning therapeutic intensity with disease severity and comorbidity profile, the framework supports precision medicine principles and avoids both undertreatment of high-risk individuals and overtreatment of those with lower disease burden (Busetto et al., 2024; Guidelines, 2025; Rubino et al., 2025; De Luca et al., 2024). This approach mirrors strategies used successfully in other chronic conditions such as hypertension, diabetes, and heart failure, thereby facilitating implementation within established clinical workflows.

The document also emphasizes the multidimensional nature of obesity. Clinical manifestations extend beyond metabolic dysfunction to include mechanical, psychological, and functional domains. Recognition of these dimensions is particularly important in older adults, in whom sarcopenia, frailty, osteoarthritis, and reduced mobility may represent dominant disease consequences (Busetto et al., 2024; Rubino et al., 2025). By prioritizing preservation of muscle function, bone mass and quality of life, the framework aligns therapeutic goals with patient-centered outcomes rather than weight reduction alone (Di Vincenzo et al., 2025).

Another key contribution is the structured integration of pharmacotherapy and metabolic surgery within a chronic disease model. Historically, pharmacologic and surgical interventions were considered escalation steps after failure of lifestyle therapy. Current evidence demonstrates that early use of effective therapies—particularly incretin-based agents and metabolic surgery in appropriately selected individuals—can modify disease trajectory, improve organ function, and reduce long-term complications (Finer et al., 2000; Torgerson et al., 2004; Jastreboff et al., 2022; Jastreboff et al., 2025; Rubino et al., 2021; Perreault et al., 2022; Wilding et al., 2021; Pi-Sunyer et al., 2015; Davies et al., 2015; le Roux et al., 2017; Lincoff et al., 2023; Packer et al., 2025; Kosiborod et al., 2024; Malhotra et al., 2024; Li et al., 2025; Blackman et al., 2016; Armstrong et al., 2016; Sanyal et al., 2025; Bliddal et al., 2024; Loomba et al., 2024). Positioning these interventions within a staged algorithm supports rational sequencing of treatment modalities and reflects modern therapeutic evidence. The relative emphasis on pharmacotherapy reflects the current strength of randomized clinical trial evidence; however, all four treatment pillars are considered equally essential within a comprehensive chronic disease model.

The statement also addresses an important translational gap between international recommendations and national implementation (Busetto et al., 2024; Guidelines, 2025; Rubino et al., 2025; De Luca et al., 2024; Grunvald et al., 2022; McGowan et al., 2025; World Health Organization, 2025). Differences in reimbursement policies, healthcare infrastructure, and resource availability often limit adoption of guideline-recommended therapies. By incorporating cost-access considerations and proposing a prioritization framework adapted to local conditions, this document provides a pragmatic pathway for translating evidence into practice. Such contextualization is essential for ensuring equity in obesity care and reducing disparities in treatment availability.

From a health-system perspective, the proposed model underscores the importance of coordinated, multidisciplinary care. Effective obesity management requires collaboration among primary care providers, specialists, surgeons, dietitians, psychologists, and rehabilitation professionals. Fragmented care pathways are associated with delayed treatment escalation, suboptimal adherence, and poorer outcomes. Structured integration across care levels can improve continuity, enhance patient engagement, and optimize long-term disease control.

This statement has several limitations. As a consensus document, some recommendations rely partly on expert agreement where high-certainty evidence is limited, particularly in areas such as optimal follow-up intervals, sequencing strategies after pharmacotherapy failure, and patient selection thresholds for surgery at lower BMI levels. Additionally, rapid therapeutic advances in obesity pharmacotherapy may necessitate periodic updates as new trial data emerge. Although this consensus emphasizes a multidisciplinary approach, the Delphi panel consisted predominantly of physicians. Allied health professionals, including dietitians and psychologists, were not formally included in the voting panel. This reflects the current structure of obesity care in Croatia, where diagnostic and pharmacological decisions are primarily physician-led. However, this represents a limitation, and future consensus processes should incorporate a broader range of healthcare professionals to enhance multidisciplinary integration. Nevertheless, use of a structured Delphi process and explicit evidence grading strengthens methodological rigor and transparency. Furthermore, economic analyses specific to Central and Eastern European healthcare systems remain limited and warrant further investigation.

Future directions in obesity management are likely to include combination pharmacotherapy, precision-targeted treatment selection based on phenotyping, integration of digital health tools for monitoring and behavioral support, and expansion of preventive strategies in earlier disease stages. Establishment of national registries and longitudinal outcome monitoring will be essential for evaluating real-world effectiveness, safety, and cost-efficiency of interventions. Continued research is also needed to refine staging systems, identify predictors of treatment response, and optimize care for special populations such as older adults and individuals with multimorbidity.

## 10 Conclusion

This position statement provides an evidence-based, consensus-driven framework for the diagnosis, staging, and management of obesity that aligns clinical practice with contemporary chronic disease principles. By integrating anthropometric assessment, functional evaluation, comorbidity profiling, and structured staging, it enables individualized treatment planning across the full spectrum of disease severity.

The proposed model emphasizes multidisciplinary care, stage-based therapy, and long-term follow-up as core elements of effective obesity management. Incorporation of pharmacological and surgical interventions within a unified therapeutic algorithm reflects current evidence demonstrating that timely, appropriately selected treatment can modify disease course, reduce complications, and improve quality of life.

Adaptation of international recommendations to national healthcare contexts represents a critical step toward equitable and practical implementation. By addressing resource variability, access constraints, and organizational factors, this framework offers a scalable approach applicable to diverse healthcare systems.

Ultimately, recognizing obesity as a chronic disease requiring structured, lifelong care is essential for improving patient outcomes and reducing the global burden of obesity-related morbidity and mortality. Implementation of integrated, stage-based management strategies has the potential to shift clinical practice from reactive treatment of complications to proactive preservation of health, function, and longevity.

This consensus framework provides a clinically actionable model for translating modern obesity science into real-world practice across healthcare systems.

## Glossary

OM: obesity medication
ADLs: Activities of daily living
AHI: Apnea–hypopnea index
ALT: Alanine aminotransferase
AST: Aspartate aminotransferase
BIA: Bioelectrical impedance analysis
BMI: Body mass index
CES: Croatian Endocrine Society
CHF: Chronic heart failure
CI: Confidence interval
CPAP: Continuous positive airway pressure
CVD: Cardiovascular disease
CVOT: Cardiovascular outcomes trial
DEXA: Dual-energy X-ray absorptiometry
EMR: Electronic medical record
EASO: European Association for the Study of Obesity
FDA: U.S. Food and Drug Administration
GI: Gastrointestinal
GLP-1: Glucagon-like peptide-1
GIP: Glucose-dependent insulinotropic polypeptide
GRADE: Grading of Recommendations Assessment, Development and Evaluation
HbA1c: Glycated hemoglobin
HDL: High-density lipoprotein cholesterol
HF: Heart failure
HFpEF: Heart failure with preserved ejection fraction
HR: Hazard ratio
ICD: International Classification of Diseases
IGT: Impaired glucose tolerance
LDL: Low-density lipoprotein cholesterol
MASLD: Metabolic dysfunction-associated steatotic liver disease
MASH: Metabolic dysfunction-associated steatohepatitis
MACE: Major adverse cardiovascular events
MI: Myocardial infarction
NASH: Non-alcoholic steatohepatitis
NEJM: New England Journal of Medicine
NICE: National Institute for Health and Care Excellence
OA: Osteoarthritis
OSA: Obstructive sleep apnea
PCOS: Polycystic ovary syndrome
RCT: Randomized controlled trial
SBP: Systolic blood pressure
T2D/T2DM: Type 2 diabetes (mellitus)
TG: Triglycerides
TSH: Thyroid-stimulating hormone
WHO: World Health Organization
WtHR: Waist-to-height ratio
